# Individual Differences in Moral Disgust Do Not Predict Utilitarian Judgments, Sexual and Pathogen Disgust Do

**DOI:** 10.1038/srep45526

**Published:** 2017-03-31

**Authors:** Michael Laakasuo, Jukka Sundvall, Marianna Drosinou

**Affiliations:** 1 University of Helsinki, Cognitive Science Unit, Faculty of Arts, Helsinki, Finland; 2 University of Sussex, School of Psychology, Pevensey Building, Falmer, BN1 9QH, United Kingdom

## Abstract

The role of emotional disgust and disgust sensitivity in moral judgment and decision-making has been debated intensively for over 20 years. Until very recently, there were two main evolutionary narratives for this rather puzzling association. One of the models suggest that it was developed through some form of group selection mechanism, where the internal norms of the groups were acting as pathogen safety mechanisms. Another model suggested that these mechanisms were developed through hygiene norms, which were piggybacking on pathogen disgust mechanisms. In this study we present another alternative, namely that this mechanism might have evolved through sexual disgust sensitivity. We note that though the role of disgust in moral judgment has been questioned recently, few studies have taken disgust sensitivity to account. We present data from a large sample (N = 1300) where we analyzed the associations between The Three Domain Disgust Scale and the most commonly used 12 moral dilemmas measuring utilitarian/deontological preferences with Structural Equation Modeling. Our results indicate that of the three domains of disgust, only sexual disgust is associated with more deontological moral preferences. We also found that pathogen disgust was associated with more utilitarian preferences. Implications of the findings are discussed.

Moral psychological disgust research has been in a slight tumult for a while. A recent meta-analysis^1^ concluded that disgust induction effects, which should amplify people’s punitive tendencies or enhance moral condemnation, are either very minimal or non-existent. Another recent review^2^ concluded that disgust and moral judgment are somehow related, however it is not clear exactly how^3^. Prior to the meta-analysis^1^, several models had been presented for the evolution of cognitive pathways that could explain how disgust-relevant information was channeled for the use of moral cognition^2^,^4^,^5^,^6^,^7^. Nonetheless, all of these models seem to be operationally dependent on individual differences in disgust sensitivity. Given that a large body of relevant disgust research has focused on individual differences in moral judgments, it is slightly surprising that experimental studies have been mostly conducted without taking this into account^1^,^2^,^3^. There seems to be only a handful of studies that have estimated the associations between disgust sensitivity and judgment of moral violations in experimental settings^3^,^8^,^9^. Yet, moral preferences, moral cognitive judgments and individual differences in disgust sensitivity appear to be intimately associated in complicated ways.

Higher disgust sensitivity specifically predicts more conservative attitudes towards abortion and gay marriage^10^, while individuals with higher disgust sensitivity have been shown to be more avoidant of moral norm violators and to judge them more harshly^11^,^12^. Previous research has also associated sexual and moral disgust with an anti-psychopathic personality trait called Honesty-Humility from the HEXACO personality inventory^13^. In addition, sexual disgust has been associated with conservative political attitudes^14^. In some studies, disgust sensitivity is associated with norm adherence^2^; while in others, it is associated with concerns for ritualistic purity, albeit this research is still relatively scarce^15^.

Human moral cognition seems to have two major moral evaluation processors that form characteristically deontological and/or utilitarian judgments with respect to moral situations^16^,^17^,^18^,^19^. *Utilitarianism* is an ethical philosophy that aims to maximize aggregate welfare (“good”) and to minimize suffering (“bad”). It is commonly juxtaposed with the *deontological* position, which states that moral rules are inviolable and do not fluctuate across situations^16^. For utilitarians, murder can be justified when the costs are outweighed by the benefits, for instance, when killing a dangerous criminal prevents further murders from taking place. Deontologists, on the other hand, think that acts are either right or wrong irrespective of their consequences. According to their position, if a moral rule is violated in one situation, it can be violated in any situation, and thus ceases being a moral rule. “Do not kill” is a typical example of a deontological rule. For a deontologist, murder always violates the fundamental moral principle which states that people (or animals) should not be treated as objects, even if this would save lives. For a utilitarian, the ends justify the means whereas for a deontologist they do not. The notion of *characteristically* deontological or utilitarian *judgments* comes from Joshua Greene^16^, highlighting that whether a person ascribes to a certain moral philosophy is dissociated from the qualities of the judgment itself.

Studies examining the effects of disgust induction on utilitarian judgment have produced conflicting results^20^,^21^,^22^ which could in part be due to the fact that these studies have not taken disgust sensitivity into account. Considering that individual differences in disgust sensitivity are relevant in moral judgment formation, it is surprising that the links between disgust sensitivity and utilitarian preferences have not been extensively investigated. Chapman & Anderson^2^,^3^ also raise a similar issue and recommend that fundamental work should be conducted by simply evaluating individual differences in disgust sensitivity towards moral cognitive stimuli that have not been intended as disgusting. Additionally, we argue that different types of disgust sensitivity should be taken into consideration. A recent model, based on extensive evolutionary theorizing^4^, differentiates between Moral, Sexual and Pathogen Disgust. The contradictory findings – that disgust sensitivity is linked to moral judgment but disgust primes are not – could be due to the fact that the disgust induction stimuli have led to a type of disgust less relevant for moral judgment. Another possibility is that, previous studies might have systematically confounded Sexual and Pathogen Disgust^4^,^23^,^24^. As far as we are aware, this is the first study to investigate the relationship between individual differences in different components of disgust and *utilitarian* moral judgments with non-disgust inducing stimuli. Previous studies, if they have included disgust sensitivity measures in their final analysis, have not investigated all of the domains of disgust^9^, or have not investigated moral dilemmas and utilitarian judgement, but images of moral violations^8^.

Thus, we decided to investigate the links between Pathogen, Sexual and Moral Disgust^24^ with respect to the most extensively studied moral cognitive process, that of utilitarian judgment formation. We found the evolutionary theoretical basis of the TDDS to be the best fit for the current study, as the connection between a specific evolved emotion and morality seems to require an evolutionary explanation. We hypothesized that Moral Disgust should be associated with more deontological or less utilitarian moral judgements, since it is operationalized as individual dislike towards moral rule-breaking. Furthermore, based on dual process model theorizing^6^,^19^,^23^, any emotional sensitivity or emotional induction effects should lead individuals towards more deontological or norm-obedient judgments and behaviors rather than utilitarian ones. We therefore did not not expect any other effects to emerge.

We used the most commonly utilized 12 moral dilemmas originally created by Greene *et al*.^25^, since these dilemmas have been shown to have excellent psychometric properties and they reliably measure the same construct^17^. Albeit several different measurements have been used to assess utilitarian moral preferences, this set of 12 dilemmas seems to be the most extensively validated one used to measure utilitarian judgments^16^,^25^. To our knowledge, this sort of fundamental analysis between the most theoretically grounded disgust measures^24^ and utilitarian preferences has not been done previously, and our research aims to fill in this existing gap (as recommended by Chapman and Anderson^2^,^3^).

## Method

### Ethics Statement

All local laws regarding ethics for social science research were followed in full. All participation was fully voluntary and participants were informed about their right to opt out at any point without penalties. All materials used and a study protocol were reviewed and approved by the University of Helsinki Ethical Review Board in Humanities and Social and Behavioral Sciences.

### Participants and Design

The data was collected in Finland and in the Netherlands between 2011 and 2014 in conjunction with several experiments that will be reported elsewhere. Parts of the data were collected online and other parts of it in laboratory conditions. Participants filled in the questionnaires in their mother tongue in randomized order. The final data set included one thousand three hundred and ninety-one respondents (N = 1391; 65% female; ~80% Finnish) with a mean age of 25.84 years (*SD* = 7.56). Participants were compensated an average of 2.5€ for their participation. In each case, participants first gave their responses to the disgust sensitivity measures after which they continued to fill in in the moral preference measures. Informed consent was obtained from all participants

## Materials

### Three Domain Disgust Scale

This scale is based on extensive evolutionary theorizing^4^ has been developed by Josh Tybur^21^. It measures three different aspects of disgust sensitivity. The 21-item scale is divided into three sub-scales of 7 items each, labeled 1) Moral Disgust, 2) Sexual Disgust and 3) Pathogen Disgust. Participants are instructed to “think about how disgusted does this make me feel?” when answering items anchored from 1: ‘not at all disgusting’ to 7: ‘very disgusting’. Example items for Moral, Sexual and Pathogen Disgust are, respectively: 1) “Shoplifting a candy bar from a convenience store”; 2) “Hearing two strangers having sex”; 3) “Stepping on dog poop”. Higher scores on all of the sub-scales indicate more disgust sensitivity. The scale does not contain reverse coded items.

### Moral preference measure

We used 12 high-conflict moral dilemmas adopted from Greene *et al*.^22^. The dilemmas are presented in Supplementary Materials. In each of the dilemmas, the participant was instructed to assume the role of the moral agent in the scenario. The moral dilemmas deal with different topics from military emergencies to trekking accidents and even situations where the agent has to consider sacrificing their own child. Each of the dilemmas described a morally ambiguous situation where the moral agent has to judge how acceptable it is to kill or injure one person in order to save multiple others (or to prevent a person from suffering before inevitable death). The utilitarian option in each dilemma has the moral agent carry the harm out with their own hands – foe example pushing a person off a footbridge in front of a trolley.

All questions were framed in the following manner: “How acceptable is it for you to do X [e.g. ‘push the bystander off the footbridge’]?”. All questions were anchored from 1: ‘not at all acceptable’ to 7: ‘totally acceptable’. By conventional standards the sacrificial dilemmas had a good inter-item reliability (Cronbach’s α = 0.87). Since Cronbach’s α is known to have psychometric problems^26^,^27^, we also calculated Tarkkonen’s ρ for the items and their internal reliability (ρ = 0.71), which indicated acceptable internal consistency as well.

### Evaluation Criteria for Structural Equation Modeling

For our Structural Equation Modeling (SEM) and Confirmatory Factor Analysis (CFA), we used the statistical programming language R and a peer-reviewed structural equation modeling library called *lavaan*^28^. Lavaan is a reliable OpenSource alternative for Mplus and provides the same model evaluation criteria. Here, we report the most common ones recommended by Kline^29^ which are: 1) *Χ*^2^, 2) The comparative fit index (CFI), 3) The root mean square error of approximation (RMSEA), and 4) Standardized root mean square residual (SRMR). We also report TLI as recommended by Byrne^30^.

*Χ*^2^ is traditionally used in CFA as a fit index and it is expected to be as close to zero as possible, thus not expected to be significant (i.e. *p*-value should be >0.05); in practice however, with sample sizes >200 it is almost always statistically significant (see refs 29 and 30 for discussion). Nonetheless, *Χ*^2^ can still be helpful in estimating fits between several models. CFI is an index with values from 0 to 1 measuring discrepancy between the hypothesized model and the actual data. CFI is not influenced by the sample size. A CFI of 0.90 is usually considered to be a passable value; however the usefulness of CFI is dependent on the complexity of the model and the available sample size. When dealing with complex models and big samples (N > 1000) values above 0.95 indicate excellent fit^31^. RMSEA is an absolute measure of a model fit, which improves as the number of variables in the model or the number of observations in the sample go up. Cut-off points of 0.01, 0.05, and 0.08 have been suggested, corresponding to excellent, good, and mediocre fits respectively^32^; confidence intervals should be used to understand the size of sampling error (upper-bound should preferable be <0.1). The SRMR indicates the difference between observed and predicted values, zero indicating perfect fit and values <0.08 are considered to indicate a good fit^33^. TLI, or Tucker-Lewis Index is a similar measure to CFI but it imposes heavier penalties for complex models; values close to 0.95 are considered to be the cut-off point for indicating good fit^33^; but for large samples with complex models, values over 0.94 indicate excellent fit^31^.

### Data Availability Statement

The data set as well as the R syntax used for the analyses presented here are available at the Open Science platform Figshare, doi:10.6084/m9.figshare.4545952.

## Results

**Figure 1:**
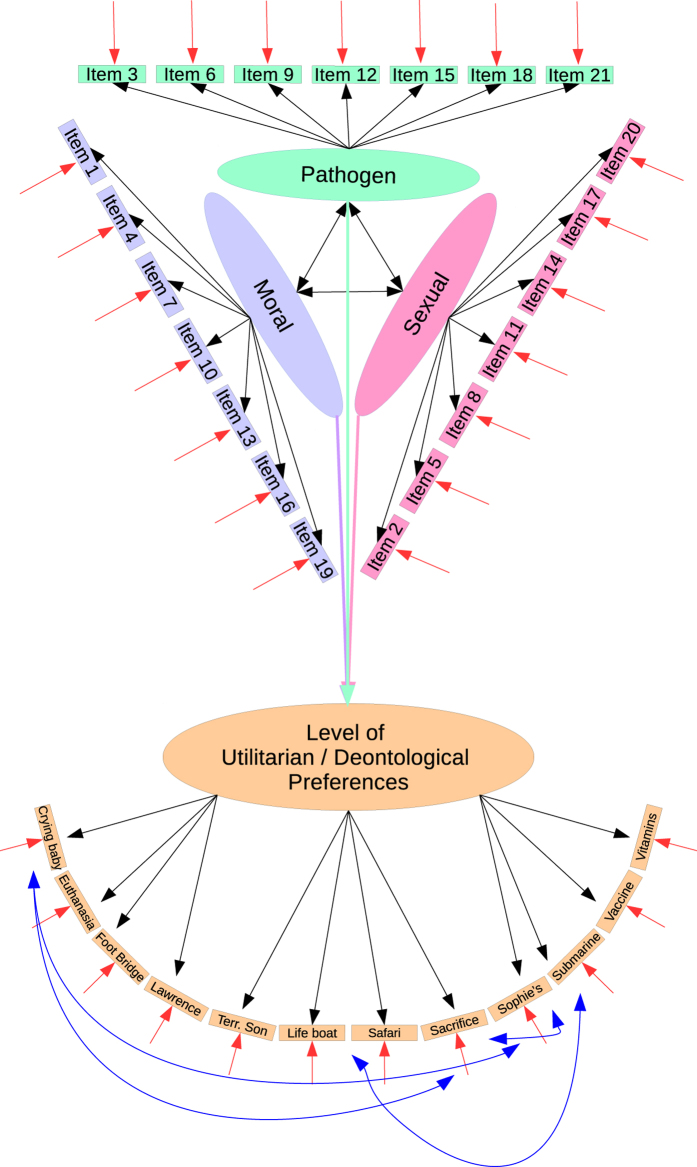
A priori model for our structural equation model. We decided to test the assumption that all disgust dimensions would be associated with the utilitarian/deontological preferences.

Our hypothesized *a priori* model is presented in Fig. 1. In our hypothesized model, we followed the recommendations of Byrne^30^ and postulated a saturated model, which would be modified if necessary. In our *a priori* model we assumed that each of the disgust sensitivity factors would be associated with Utilitarian/Deontological preferences (See Fig. 1 for our hypothesized model). However, before building the structural equation, we first ran a Confirmatory Factor Analyses for both of our constructs, which we then combined to form the model portrayed in Fig. 1.

### Confirmatory Factor Analyses

#### Three Domain Disgust Scale

For this scale we first built the standard model suggested by previous research^24^ (see Table 1 for listing of items). We then ran a robust MLM estimated (i.e. Satorra-Bentler corrected) CFA for this model. Given that our sample size was big, this baseline model did not fit the data adequately (*χ*^*2*^_(186)_: 1174.17, CFI: 0.90, TLI: 0.89, RMSEA: 0.06, 90%CI: [0.06, 0.07], SRMR 0.05; c.f. ref. 28). This seems to replicate^34^ (see Study 2), who also reported relatively weak model fit indices in their data. Due to these reasons, we proceeded with model modifications aiming specifically for the CFI cut-off point of 0.95, which indicates better than good fit for complex models in large samples^31^. Model modifications are usually done to improve the model fit with data^30^, since sub-optimally fitting models might give rise to inaccurate parameter estimates (e.g. covariances between factors; see ref. 29).

**Figure 2:**
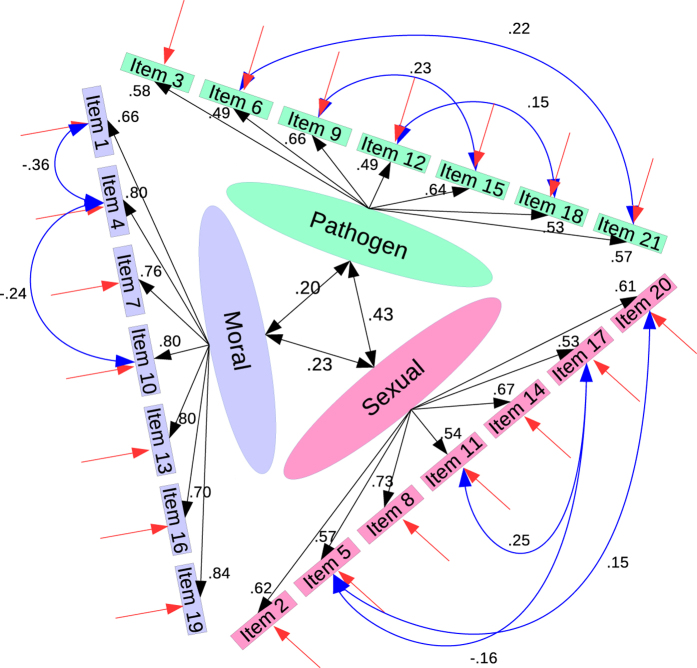
Final model for Three Domain Disgust Scale (TDDS) after modifications. All loadings and error covariances presented in standardized forms. This final model for TDDS had a very good fit with the data (SBχ^2^_(178)_) = 735.22, p < 0.05; CFI = 0.947, TLI = 0.938; RMSEA = 0.047, [0.044, 0.051]; SRMR = 0.049).

We modified the model step-by-step based on the recommended model modification indices. We always chose the largest suggested modification, if the suggested modification was such that there was error covariance to be added between two items measuring the same latent variable. No cross loadings were added, since all cross loading suggestions made by the software were substantially unwarranted (e.g. pathogen disgust associated with the item asking for the level of disgust experienced when somebody has sexual fantasies about one-self). For the full list of added error covariances and associated statistics, see Table 2. For the final version of the model, see Fig. 2. The associations between the error terms also made substantial sense (e.g. Items 6 and 21 relate to open wounds; Items 1 and 4 are about stealing; Items 11 and 17 make the respondent a target for somebody else’s sexual interest, etc).

#### Moral Preferences Measure

**Figure 3:**
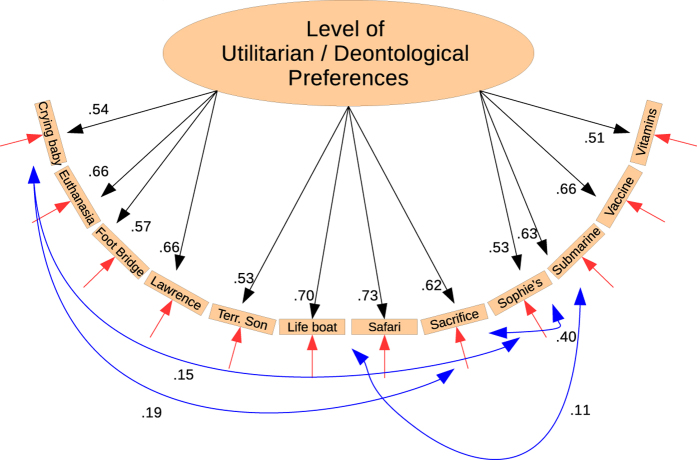
Results of Confirmatory Factor Analysis for The Moral Preference Measure consisting of 12 moral dilemmas. This model was adapted directly from Laakasuo and Sundvall (2016) and was not modified, since it fit the model very well (SBχ^2^_(66)_ = 240.187, p < 0.05; CFI: 0.965, TLI: 0.954; RMSEA: 0.052, [0.046, 0.058]; SRMR: 0.032).

In a recently published paper^17^, Laakasuo & Sundvall demonstrated that all the 12 items most commonly used conform to a unidimensional model. However, the model which the authors presented was dependent on controlling for error covariances between three dilemmas dealing with sacrificing children and another error covariance between two dilemmas set in a maritime environment. We fitted this MLM estimated model with our data. We found the fit to be excellent, so we did not proceed with any model modifications. The measurement model and associated statistics are presented in Fig. 3.

### Structural Equation Model

**Figure 4:**
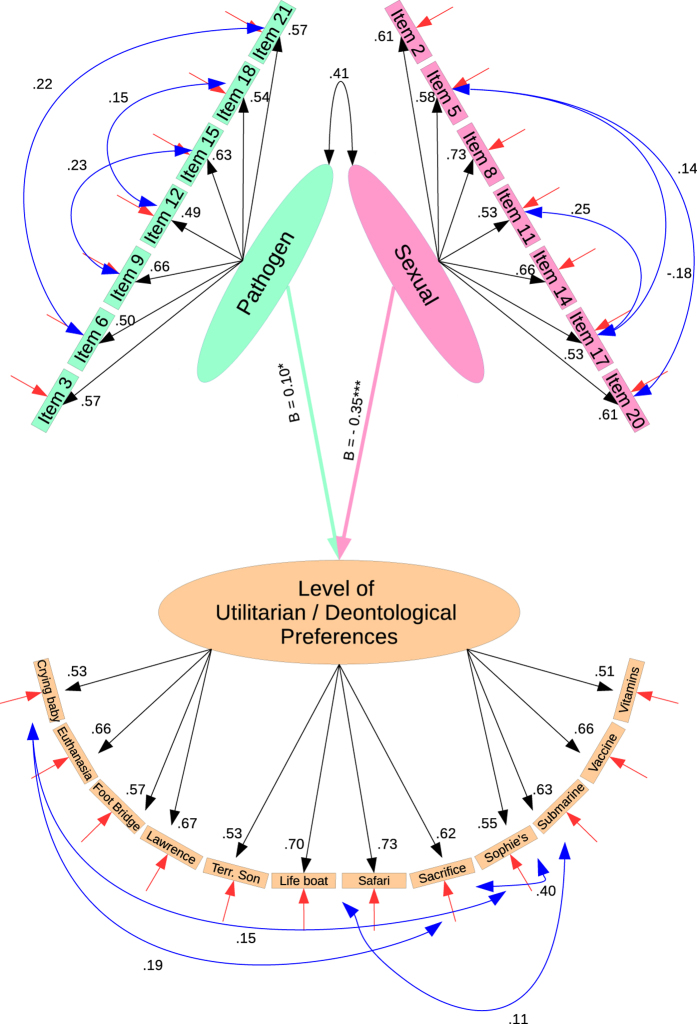
Final results of our Structural Equation Model analysis where two disgust factors have been regressed on Utilitarian preferences. The regression coefficients are unstandardized values, while all other values are standardized factor loadings or error covariances. The final model fitted the data moderately well (χ^2^_(286)_ = 921.28, p < 0.05; CFI: 0.941, TLI: 0.933; RMSEA: 0.04, [0.037, 0.043]; SRMR: 0.039); *p = < 0.05, ***p < 0.001)

After we prepared our latent constructs we put them together and regressed all the Disgust dimensions on our DV (Utilitarian/Deontological Preferences Measure). The model was fitted using robust MLM estimation (i.e. Satorra-Bentler corrections). In the resulting analysis, we found that the Moral Disgust dimension had no association with our DV (B = −0.03, *Z* = −1.21, *p* = n.s.). Unexpectedly, Pathogen Disgust was associated with more Utilitarian preferences (i.e. it had a positive association with our DV: B = 0.12, *Z* = 2.42, *p* < 0.05) and Sexual Disgust was associated with more deontological preferences/*negatively* with utilitarian preferences (B = −0.34, *Z* = −8.44, *p* < 0.001). We therefore continued our analysis by removing the regression between Moral Disgust and our DV, along with the items which loaded on to the construct. The resulting model had good fit with the data (see Fig. 4 for full statistics). See supplementary materials for exploratory analyses.

## Discussion

We studied associations between three types of disgust on deontological/utilitarian preferences. Our results suggest that sexual disgust is related negatively with utilitarian preferences, while pathogen disgust is related positively with utilitarian preferences (if the latent factors are allowed to correlate, see Supplementary Materials). While few previous studies on individual variation in disgust sensitivity and utilitarianism have been conducted, none of them have estimated this association with a large sample without experimental manipulations. Our findings indicate that there has indeed been a gap in the existing literature regarding how different types of individual differences in disgust sensitivity are related to deontological/utilitarian moral judgments.

In addition, our results indicate that individual variation in Sexual Disgust sensitivity is the strongest predictor of deontological or non-utilitarian preferences compared to the other two disgust dimensions, as measured by the Three Domain Disgust scale. Furthermore, there seems to be a smaller effect for Pathogen Disgust in predicting the opposite effect; Pathogen Disgust sensitivity predicts utilitarian responses positively. However, this effect is contingent on the fact that the two disgust dimensions share a relatively large covariance; if the two dimensions are treated orthogonally, as in normal regression analysis, only Sexual Disgust remains statistically significant (see Supplementary Materials).

Several studies in moral psychology have suggested a specific mapping between certain emotions and different types of moral violations, but a recent review^35^ calls this into question, noting that there is much overlap in these associations. Specifically, anger has been traditionally associated with the moral domain of harm, whereas disgust has been associated with the domain of purity. Our results are in line with the review’s suggestion^35^ that there is no exclusive mapping between these emotions and moral domains. Here, individual sensitivity to subtypes of disgust was associated with differences in judgment of harm caused (arguably) for “the greater good”.

Theoretical arguments for these findings can only be speculated upon, since one would have expected moral disgust to have been the most important predictor for deontological preferences. Although the evolutionary origins of moral disgust are not completely clear^4^,^7^,^23^, the results can still be evaluated from the classical kin-selection and sexual selection model perspectives. If pathogens are something that are contagious and spread throughout one’s immediate environment (e.g. tribe or one’s extended family), the killing of possibly diseased kin or tribe members makes plausible sense from specific perspectives (see ref. 36 for a review on killing off of one’s kin in tribal societies). In these situations, Pathogen Disgust should be expected to function in a utilitarian manner – i.e. it is better to lose one sick brother than three (see ref. 37 for a similar conclusion with regards to utilitarian reasoning in general).

On the other hand, if we look at sexual selection theories and empirical findings related to sexual selection, we have more or less established that kindness, fairness, trustworthiness and loyalty are sought-after features in long-term partners^38^ (see ref. 39 for a review). Furthermore sexual disgust has evolved through pressures stemming from sexual selection^4^,^24^. Thus, with the wisdom of hindsight, it is not surprising that sexual selection should be associated with moral judgment preferences for respecting moral rules. After all, a fully utilitarian partner, who has no intrinsic regard for the value of human life *an sich*, would not be a preferable partner or an appealing parent.

In a critique of the moral dilemma paradigm, Kahane^40^ notes that the utilitarianism measured by the moral dilemmas does not necessarily reflect concern for aggregate welfare or impartiality, the two most important factors in the philosophy of utilitarianism (see also ref. 41 for a similar critique). Some of the commonly used dilemmas are presented with the moral agent as one of the people to be saved, and in others the person to be sacrificed is at least partially to blame for the dilemma situation. Kahane suggests that a “utilitarian” response to a dilemma like this may simply be an indicator of concern for one’s own well-being and/or willingness to kill a person who can be blamed for the difficult situation. Utilitarian responses to high-conflict dilemmas have also been associated with “explicit amoral and self-centered judgments” in other situations^42^.

Given these findings, in addition to studies linking utilitarian preferences with psychopathic tendencies^43^ and dysfunctional emotional processing^44^, a negative link between Sexual Disgust and these moral judgments should not have been unexpected. As the main function of Sexual Disgust is to reject ill-suited mates from a subjective perspective^4^, there are (at least) two mutually non-exclusive ways to explain the connection.

First, it could be that utilitarian individuals, being possibly more psychopathic or faulty in their emotional processing, also have more lax filters when it comes to rejecting possible mates. Psychopathic personality traits are associated with a greater number of lifetime sexual partners and greater incidence of long-term relationship breakdown^45^. Second, the effect may reflect something in the more deontological individuals’ preferences. In our study, we posed all dilemmas in the same way, asking our participants to indicate to what extent they find it acceptable to sacrifice one to save many. A person who has stricter filters for suitable mates, who would find a coldly utilitarian partner not very appealing, would likely also refrain (or at least claim to refrain) from such cold actions themselves. Indeed, actual sex differences have been reported to this end; deontologists are considered to be more trustworthy and likable than utilitarians, and this effect is bigger for women than for men, which supports our theorizing and fits with our results (see ref. 46).

It has been suggested^18^ that a method called Process Dissociation should be used to separate utilitarian and deontological moral preferences from one another. Otherwise it is impossible to dissociate between e.g. increased utilitarian preferences or decreased deontological preferences. Our approach does not take this into account, since the paper drawing attention to this theoretical issue^18^ was published after most of our data was collected. We have juxtaposed these preferences into a single measure which was anchored from “not at all acceptable to kill” to “very acceptable to kill”. In our model it is assumed implicitly that if it is “not at all acceptable” to kill a person to save many, this is due to people following an implicit rule: killing is never acceptable.

Like all studies, this study also suffers from standard set of limitations facing laboratory or internet questionnaires in general. Our respondents are not a randomly collected sample from a more general population (e.g. our sample could have a self-selection bias of containing more curious and patient individuals than what could be expected by chance). Furthermore, they are much younger than the Finnish population average. As is also the case, survey studies utilizing self-report measures might be biased by a mixture of positive response biases and demand characteristics. However, all the precautionary measures to minimize these were taken by informing the participants that the questionnaires are anonymous and that they are not being screened as individuals or for clinical purposes/ profiling. And as a last caveat, our results might only be applicable for moral judgments, but not for moral actions^47^,^48^; this would need to be clarified by future research.

Future studies concentrating on elucidating the associations between disgust and morality should pay more careful attention towards the construct that is commonly labeled “physical disgust”. As previous studies have already shown, it clearly breaks down into two separate components which also seem to have quite different associations with the machinery that produces moral cognition and judgments. According to Chapman & Andersson^2^, most of the research effort put into studying the links between moral cognition and disgust has focused on contrasting “physical disgust” with moral disgust, and as we have seen, this approach has not produced sufficiently reliable results^1^. Future studies investigating the complicated associations between disgust and moral reasoning would also benefit from using extensively validated and theoretically grounded measures, rather than *ad hoc* measures devised for each experiment separately. At the time of writing, utilitarianism is operationalized with several different measures. Sometimes only a single item is used (most commonly the standard trolley dilemma; e.g ref. 49), which does not seem to be a warranted approach^17^. Furthermore, sometimes utilitarianism is operationalized with a non-standardized set of dilemmas which are only used once in a single study (e.g. refs 9,41 and 50), making it challenging to compare results produced by different studies.

## Conclusion

Our study successfully fills in an existing gap in the literature by estimating the direct effects between three domains of disgust sensitivity and utilitarian preferences. Our results suggest that Sexual Disgust sensitivity is associated with more deontological preferences when estimated with a standard-set of validated dilemmas^25^ (see also ref. 17). Thus, our results support the claim that the emotion of disgust is not exclusively associated with moral judgments of purity violations, as suggested by ref. 35. Furthermore, if the latent factors are allowed to correlate, Pathogen Disgust is associated with more utilitarian preferences, further bolstering the argument made previously by other research^4^ that “physical disgust” should be divided into Sexual and Pathogen Disgust.

## Additional Information

**How to cite this article:** Laakasuo, M. *et al*. Individual Differences in Moral Disgust Do Not Predict Utilitarian Judgments, Sexual and Pathogen Disgust Do. *Sci. Rep.* **7**, 45526; doi: 10.1038/srep45526 (2017).

**Publisher's note:** Springer Nature remains neutral with regard to jurisdictional claims in published maps and institutional affiliations.

## Supplementary Material

Supplementary Materials

## Figures and Tables

**Table 1:** Listing of the items in Three Domain Disgust Scale.

Item number	Item Content	Sub-scale
1	Shoplifting a candy bar from a convenience store	M
2	Hearing two strangers having sex	S
3	Stepping on dog poop	P
4	Stealing from a neighbor	M
5	Performing oral sex	S
6	Sitting next to someone who has red sores on their arm	P
7	A student cheating to get good grades	M
8	Watching a pornographic video	S
9	Shaking hands with a stranger who has sweaty palms	P
10	Deceiving a friend	M
11	Finding out that someone you do not like has sexual fantasies about you	S
12	Seeing some mold on old leftovers in your refrigerator	P
13	Forging someone’s signature on a legal document	M
14	Bringing someone you just met back to your room to have sex	S
15	Standing close to a person who has body odor	P
16	Cutting to the front of a line to purchase the last few tickets to a show	M
17	A stranger of the opposite sex intentionally rubbing your thigh in an elevator	S
18	Seeing a cockroach run across the floor	P
19	Intentionally lying during a business transaction	M
20	Having anal sex with someone of the opposite sex	S
21	Accidentally touching a person’s bloody cut	P
Notes: Three Domain Disgust Scale (see ref. 24 for original publication); M: Moral Disgust; S: Sexual Disgust; P: Pathogen Disgust		

**Table 2:** Pathway of Model Modifications used to correct Three Domain Disgust scale.

	Modification	Suggested MI	SBχ^2^	df	Δχ^2^	CFI/TLI	RMSEA & 90% CI	SRMR
Baseline	—	—	1174.17	186		0.907/0.891	0.062[0.059, 0.065]	0.053
Model 1	Item 1 ~~ Item 4	124.83	1070.46	185	104	0.916/0.905	0.059[0.055, 0.062]	0.053
Model 2	Item 11 ~~ Item 17	101.54	979.85	184	91	0.925/0.914	0.056[0.053, 0.059]	0.054
Model 3	Item 6 ~~ Item 21	71.80	917.77	183	62	0.931/0.920	0.054[0.050, 0.054]	0.053
Model 4	Item 4 ~~ Item 10	64.51	860.41	182	57	0.936/0.926	0.052[0.048, 0.055]	0.052
Model 5	Item 9 ~~ Item 15	53.95	814.58	181	50	0.940/0.931	0.050[ 0.047, 0.054 ]	0.052
Model 6	Item 5 ~~ Item 17	36.21	778.89	180	36	0.943/0.934	0.049 [0.046, 0.052]	0.051
Model 7	Item 12 ~~ Item 18	25.93	755.88	179	23	0.946/0.936	0.048[0.045, 0.052]	0.051
Model 8	Item 5 ~~ Item 20	24.52	735.22	178	22	0.947/0.938	0.047[0.044, 0.051]	0.049
Note: ~~ means added error covariance. Each step of the modifications improved the model fit statistically significantly (*p* < 0.001).								
